# Macrophage activation syndrome triggered by systemic lupus erythematosus flare: successful treatment with a combination of dexamethasone sodium phosphate, intravenous immunoglobulin, and cyclosporine: a case report

**DOI:** 10.1186/s13256-021-03072-1

**Published:** 2021-10-07

**Authors:** Wesam Gouda, Faisal Alsaqabi, Abdelhfeez Moshrif, Awad S. Abbas, Tarek M. Abdel-Aziz, Md Asiful Islam

**Affiliations:** 1 Department of Rheumatology, Faculty of Medicine, Al Azhar University, Assiut, Egypt; 2 Department of Rheumatology, Al‐Sabah Hospital, Kuwait, Kuwait; 3 Department of Haematology, School of Medical Sciences, Universiti Sains Malaysia, Kubang Kerian, Kelantan Malaysia

**Keywords:** Hemophagocytic lymphohistiocytosis (HLH), Intravenous immunoglobulin (IVIG), Macrophage activation syndrome (MAS), Systemic lupus erythematosus (SLE), Cyclosporine, Dexamethasone sodium phosphate (DSP)

## Abstract

**Background:**

Macrophage activation syndrome is classified as a secondary form of hemophagocytic lymphohistiocytosis. It is a hyperinflammatory complication observed to be comorbid with a variety of autoimmune diseases, including adult-onset Still’s disease and systemic juvenile idiopathic arthritis. Macrophage activation syndrome is less commonly detected in adult patients with systemic lupus erythematosus, which, if untreated, can be fatal, though determining the optimum treatment strategy is still a challenge.

**Case presentation:**

Herein, we report a case of macrophage activation syndrome in a 33-year-old Egyptian female as an unusual complication of a systemic lupus erythematosus flare in adult patients. Our patient was initially treated with a combination of intravenous methylprednisolone pulse therapy and intravenous immunoglobulin therapy, which was followed by a course of oral prednisolone and oral cyclosporine with little response. Switching from oral prednisone to intravenous dexamethasone sodium phosphate showed a more favorable clinical and biochemical response.

**Conclusion:**

Macrophage activation syndrome is less commonly detected in adult patients with systemic lupus erythematosus. Our case demonstrates that dexamethasone sodium phosphate can be a successful alternative treatment for patients with systemic lupus erythematosus complicated by macrophage activation syndrome in whom the response to pulse methylprednisolone was inadequate to manage their illness, proving to be remarkably effective in a relatively short time frame.

## Background

Macrophage activation syndrome (MAS) is a life-threatening condition characterized by cytopenia, high fever, liver insufficiency, and coagulopathy. Excessive activation and proliferation of T lymphocytes and macrophages or histiocytes lead to extensive hemophagocytosis in the bone marrow and a cytokine storm [1]. MAS, also known as an autoimmune-associated hemophagocytic syndrome (AAHS), is considered as a type of secondary hemophagocytic lymphohistiocytosis (sHLH) in patients with autoimmune or autoinflammatory disorders [2]. It accounts for 7.0–15.3% of sHLH [3, 4]. As a complication of systemic lupus erythematosus (SLE) in adults, it is relatively uncommon. The occurrence of MAS in SLE has been reported to range from 0.9% to 4.6% [5].

Because MAS and SLE share clinical and laboratory features, which include fever, cytopenia, and splenomegaly, distinguishing MAS from SLE flare has been challenging. Following multiple-organ failure, if MAS is left untreated or even undetected, the mortality rates can rise to 11% and 42% in pediatric and adult patients, respectively [6, 7].

Several treatment options for MAS in SLE have recently been proposed: high-dose corticosteroid treatment including pulse methylprednisolone (mPSL) is the suggested initial treatment of choice in MAS, and second-line agents include cyclosporine, azathioprine, tacrolimus, etoposide, thalidomide, or anakinra [8, 9]

Here, we are reporting a case of SLE flare complicated with MAS in a middle-aged female, who was successfully treated with dexamethasone sodium phosphate (DSP), intravenous immunoglobulin therapy (IVIG), and oral cyclosporine after a poor response to mPSL. We hypothesized that DSP could be a successful alternative treatment for MAS patients in whom the response to pulse mPSL was inadequate to manage their illness.

## Case description

A 33-year-old Egyptian female with a 4-week history of fever, abdominal pain, malaise, and progressive fatigue was referred for hospital admission after being discovered to have pancytopenia. She had a 5-year history of SLE and antiphospholipid syndrome (APS) featuring arthritis, oral ulcers, leukopenia, positive antinuclear antibodies (ANA), Anti-double stranded DNA (Anti-dsDNA), recurrent venous thromboembolism (VTE), miscarriages, and antiphospholipid antibodies, confirming the diagnosis of SLE (ACR 5/11 and SLICC 5/13) with APS [10, 11]. She had no previous history of skin rash, For the previous 8 months, she had been taking hydroxychloroquine (400 mg/day), prednisolone (7.5 mg/day), and warfarin (5 mg/day). Upon admission, she was febrile with a maximum temperature of 38.6 °C, pulse was 107 beats per minute, and she had otherwise normal vital signs. She had a height of 175 cm and weighed 80 kg. Physical examination revealed an enlarged liver of 3 cm and a spleen of 4 cm palpable below the costal margin, while examination of the respiratory system, nervous system, and precordium was normal. Laboratory studies showed pancytopenia, reticulocytosis, hyponatremia, liver dysfunction, and proteinuria. Further workup showed that she had marked elevated d-dimer, ferritin, lactate dehydrogenase (LDH), and triglycerides, prolonged activated partial thromboplastin time (aPTT), decreased complement levels (C3), and elevated erythrocyte sedimentation rate (ESR), with positive direct antiglobulin (Coombs) test. A 24-hour urine protein test demonstrated proteinuria in the subnephrotic range (0.54 g/24 hours). Abdominal ultrasonography confirmed hepatosplenomegaly, while chest X-ray and echocardiography were normal. The SLE Disease Activity Index (SLEDAI) score was 11, indicating a moderate flare.

Our differential diagnosis was taken into consideration: MAS, SLE flare-up, viral infection, sepsis, leukemia, and lymphoma. The diagnosis, however, was difficult in part owing to strong similarities between MAS, SLE flare, and sepsis. Repeated blood, urine cultures, and viral panels for SARS-CoV-2, hepatitis B and C viruses, Epstein–Barr virus, herpes simplex virus, coxsackievirus, cytomegalovirus, human immunodeficiency virus, and parvovirus B19 were all shown to be negative. Renal biopsy was considered. However, due to the patient’s severe thrombocytopenia, it was postponed.

Based on the laboratory and clinical findings in accordance with the HLH diagnostic criteria, a diagnosis of MAS secondary to SLE flare was made. Intravenous methylprednisolone (mPSL) 1 g/day for 3 days followed by oral prednisone 60 mg daily and IVIG 2 g/kg over 5 days was started. Subsequently, oral cyclosporine 300 mg daily was started. The fever, abdominal pain, malaise, and fatigue were partially resolved while her counts were a little improved 7 days after starting treatment. Hence, we decided to switch from oral prednisolone to intravenous DSP 9 mg/day. Within 4 days, the platelet, white blood cell (WBC) counts, hemoglobin, inflammatory indices, LDH, and ferritin levels all eventually returned to normal (Table 1).

**Table 1:** Laboratory data upon admission, discharge, and 6 weeks after discharge

	Reference range	Admission	After mPSL	After DEX	6 weeks after discharge
White blood cell count (10^9^/L)	4.5–11.0	0.72	1.12	4.08	6.01
Lymphocyte (10^9^/L)	1.0–4.0	0.50	0.59	0.71	1.91
Hemoglobin (g/L)	121–151	77	79	93	101
Hematocrit (L/L)	0.36–0.46	0.21	0.21	0.27	0.36
Platelets (10^9^/L)	150–400	22	29	113	287
Reticulocyte count (%)	0.5–1.5	4.47	–	–	–
Na (mmol/L)	138–145	130	131	136	138
K (mmol/L)	3.6–4.8	4.5	4.6	4.6	4.1
Creatinine (mg/dL)	0.46–0.79	0.51	0.50	0.50	0.51
ALT (U/L)	7–23	46	44	23	20
AST (U/L)	13–30	51	48	21	22
Triglyceride (mmol/L)	1.8–2.2	4.13	4.01	2.1	1.6
LDH (U/L)	124–222	1260	990	250	245
Haptoglobin (mg/dL)	41–165	21	–	–	–
Albumin (g/L)	35–55	29.8	30.2	34.1	38.8
Ferritin (ng/mL)	6.2–138	9560	8580	550	290
Fibrinogen (g/L)	2–4	3.5	3.5	3.4	3.5
d-dimer (μg/L)	< 250	4766	2255	430	102
aPTT (seconds)	30–40	123.3	91.5	62.5	42
PT (seconds)	10–14	25.1	22.3	14.3	14.1
CRP (mg/dL)	< 10	68.8	48	18	0.2
ESR (mm/hour)	22	62	51	26	29
24-hour urine protein (g/day)	0.0–0.16	0.54	–	0.16	0.15
Direct antiglobulin test	–	Positive	–	–	–
Indirect antiglobulin test	–	Negative	–	–	–
C3 (g/L)	0.90–1.80	0.62	–	–	–
C4 (g/L)	0.10–0.40	0.17	–	–	–
Anti-dsDNA antibody	< 1: 10	1:40	–	–	–
Antinuclear antibody	< 1: 40	1: 640 (speckled)	–	–	–
Anticardiolipin antibody (U/mL)	< 10	27	–	–	–
Lupus anticoagulant	< 1.3	1.71	–	–	–

*ALT* Alanine transaminase, *AST* Aspartate transaminase, *LDH* Lactate dehydrogenase, *aPTT* Partial thromboplastin time, *PT* prothrombin time, *CRP* C-reactive protein

**Fig. 1:**
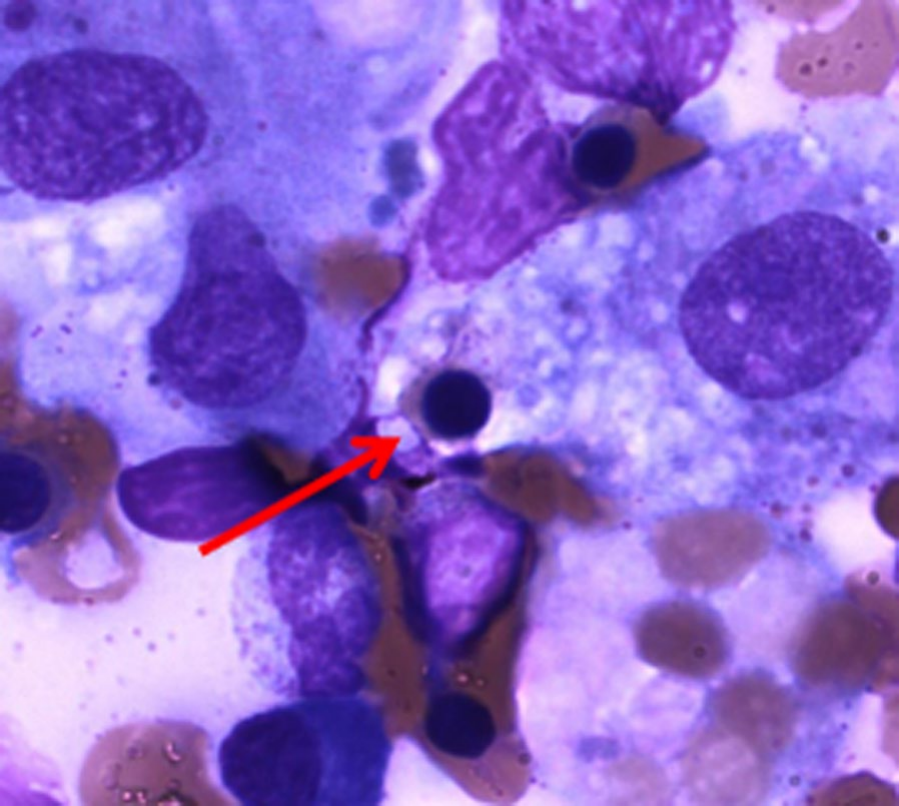
Bone marrow biopsy showing focal evidence of hemophagocytosis (arrow)

Afterward, following the late acceptance of her husband, a bone marrow biopsy was performed 7 days after starting the treatment with mPSL, which showed focal evidence of hemophagocytosis (Fig. 1). The patient was discharged without symptoms, with monthly follow-up of complete blood count, LDH, ferritin, 24-hour urine protein, anti-dsDNA antibody, and complement levels. Oral prednisone was gradually tapered to 20 mg daily, and oral cyclosporine of 300 mg daily was continued. One year after treatment, the patient remains symptom-free without any potential adverse effects of the adherent medications, and the patient-reported outcome shows patient satisfaction.

## Discussion

HLH is a rare hematologic condition that can be divided into two types: familial (primary) and acquired (secondary) HLH. sHLH is seen in a heterogeneous group of diseases including infections, malignancies, hematological disorders, and autoimmune diseases [12]. sHLH related to rheumatic diseases has been referred to as MAS since it was first reported as a complication of sJIA in 1985 by Hadchouel *et al.* [13]. The clinical characteristics of MAS-associated SLE and active SLE are very similar, making MAS diagnosis difficult. With a sensitivity and specificity of about 100%, hyperferritinemia is considered as the chief significant parameter for distinguishing between both [14].

The 2004 proposed diagnostic criteria for HLH (Table 2) serve as a useful diagnostic tool [15]. The HScore (Table 3) may be applied to determine the probability of getting sHLH in adult patients [16].

**Table 2:** Diagnostic criteria of HLH—2004

1. Fever of 38.5 °C or more
2. Splenomegaly
3. Cytopenia (affecting at least two cell lineages in the peripheral blood)
∙ Hemoglobin < 9 g/dL
∙ Platelets < 100,000/μL
∙ Absolute neutrophil count < 1000/μL
4. Hypertriglyceridemia (fasting triglycerides > 265 mg/dL) and/or hypofibrinogenemia (fibrinogen < 150 mg/dL)
5. Hemophagocytosis in bone marrow, spleen, lymph nodes, or liver
6. Low or absent natural-killer-cell activity
7. Ferritin > 500 ng/mL
8. Increased soluble CD25 concentration (alpha chain of soluble interleukin-2 receptor)
The diagnosis of HLH requires the presence of five of the above criteria

*HLH *hemophagocytic lymphohistiocytosis

**Table 3:** HScore

Parameter	No. of points (criteria for scoring)
Known underlying immunosuppression	0 (no) or 18 (yes)
Temperature (°C)	0 (< 38.4), 33 (38.4–39.4), or 49 (> 39.4)
Organomegaly	0 (no), 23 (hepatomegaly or splenomegaly), or 38 (hepatomegaly and splenomegaly)
Number of cytopenias	0 (one lineage), 24 (two lineages), or 34 (three lineages)
Ferritin (ng/mL)	0 (< 2000), 35 (2000–6000), or 50 (> 6000)
Triglyceride (mmol/L)	0 (< 1.5), 44 (1.5–4), or 64 (> 4)
Fibrinogen (g/L)	0 (> 2.5) or 30 (< 2.5)
AST, U/L	0 (< 30) or 19 (> 30)
Hemophagocytosis features on bone marrow	0 (no) or 35 (yes)

*AST* Aspartate transaminase

In secondary HLH, and MAS in patients with SLE in particular, there is no consensus treatment guideline for management. High-dose glucocorticoids, followed by second-line therapies such as cyclosporine, cyclophosphamide, anakinra, etoposide, and IVIG for severe or refractory cases, and plasma exchange in patients with life-threatening MAS were reported [17].

Corticosteroids inhibit the inflammatory function of macrophages through glucocorticoid receptors. Since the glucocorticoid receptors are located in the cytoplasm, effective delivery of corticosteroids into the cytoplasm can enhance their therapeutic effect on macrophages. Liposome-bound dexamethasone is effectively used by macrophages through phagocytosis. As a result, a large amount of DSP remains in the cytoplasm [18]. The reticuloendothelial system and some inflammatory cells, including macrophages, utilize DSP, a liposome-incorporated dexamethasone, which is much more efficient than free corticosteroids. Therefore, DSP has stronger anti-inflammatory activity [19].

Our case was presented with unremitting fever, progressive pancytopenia, hepatosplenomegaly, hyperferritinemia, hypertriglyceridemia, hypofibrinogenemia, and hyponatremia, with the diagnosis of SLE flare-associated MAS being made as she met the diagnostic criteria for HLH-2004 (5/8) prior to bone marrow examination. Our patient additionally had an HScore of 271, suggesting a greater than 99% probability of having sHLH/MAS [16].

Our patient was initially treated with intravenous mPSL pulse therapy, which was followed by a course of oral prednisolone, IVIG therapy, and oral cyclosporin, with the goal of treating MAS and inducting remission of SLE activity. However, after 7 days of treatment, the response to these drugs was not sufficient to manage their diseases. Subsequently, within 4 days after switching from prednisolone to intravenous DSP, the platelet, WBC counts, inflammatory markers, LDH, and ferritin levels dramatically returned to normal. To our knowledge, this is the first case report describing the successful use of DSP in a refractory case of MAS in adult SLE. The use of DSP has been driven by lessons observed in refractory cases of pediatrics MAS, considering the HLH-2004 dexamethasone, cyclosporine, and etoposide treatment regimen [20]. Furthermore, in two reports, dexamethasone palmitate (DP), a liposome-incorporated form of dexamethasone, was effective in a pediatric case of MAS associated with s-JIA, juvenile SLE (jSLE), and Kawasaki disease that was resistant to pulse methylprednisolone [21, 22]. From these findings, we hypothesized that DSP directly activated macrophages that could produce more effective anti-inflammation compared with other corticosteroids.

This study has some limitations. Because it is not based on systematic studies, it lacks the potential to generalize.

## Conclusion

MAS should be included in the differential diagnosis of SLE patients with persistent fever and pancytopenia. Physicians ought to have a high level of suspicion for diagnosing MAS in the setting of relevant clinical symptoms and signs. Early and proper treatment is crucial to prevent the high mortality rate. Rather than renal abnormalities, anti-dsDNA antibody high titer, and complement levels, hyperferritinemia and hypertriglyceridemia are regarded as the most important parameters for differentiating between MAS and SLE flare.

Our case demonstrates that DSP can be a successful alternative treatment for MAS patients in whom the response to pulse mPSL was inadequate to manage their illness, proving to be remarkably effective in a relatively short time frame. In the future, larger trials are required to determine the value and exact mechanism of DSP in MAS patients.

## Data Availability

The original data generated and analyzed for this study are included in the published article. Further inquiries can be directed to the corresponding author.

## Abbreviations

MAS: Macrophage activation syndrome
AAHS: Autoimmune-associated hemophagocytic syndrome
sHLH: Secondary hemophagocytic lymphohistiocytosis
SLE: Systemic lupus erythematosus
DSP: Dexamethasone sodium phosphate
APS: Antiphospholipid syndrome
VTE: Venous thromboembolism
ACR: American College of Rheumatology
SLICC: The Systemic Lupus Collaborating Clinics
LDH: Lactate dehydrogenase
SLEDAI: Systemic Lupus Erythematosus Disease Activity Index
mPSL: Methylprednisolone
IVIG: Intravenous immunoglobulin
DSP: Dexamethasone palmitate
jSLE: Juvenile systemic lupus erythematosus

## References

[1] Dhote R, Simon J, Papo T, Detournay B, Sailler L, Andre MH, et al. Reactive hemophagocytic syndrome in adult systemic disease: report of twenty-six cases and literature review. Arthritis Rheum. 2003;49(5):633–9. 10.1002/art.11368 14558048

[2] Rosado FG, Kim AS. Hemophagocytic lymphohistiocytosis: an update on diagnosis and pathogenesis. Am J Clin Pathol. 2013;139(6):713–27. 10.1309/AJCP4ZDKJ4ICOUAT 23690113

[3] Li F, Yang Y, Jin F, Dehoedt C, Rao J, Zhou Y, et al. Clinical characteristics and prognostic factors of adult hemophagocytic syndrome patients: a retrospective study of increasing awareness of a disease from a single-center in China. Orphanet J Rare Dis. 2015;10(1):1–9. 10.1186/s13023-015-0224-y PMC4355377 25757854

[4] Li J, Wang Q, Zheng W, Ma J, Zhang W, Wang W, et al. Hemophagocytic lymphohistiocytosis: clinical analysis of 103 adult patients. Medicine. 2014;93(2):100–5. 10.1097/MD.0000000000000022 PMC4616310 24646466

[5] Vilaiyuk S, Sirachainan N, Wanitkun S, Pirojsakul K, Vaewpanich J. Recurrent macrophage activation syndrome as the primary manifestation in systemic lupus erythematosus and the benefit of serial ferritin measurements: a case-based review. Clin Rheumatol. 2013;32(6):899–904. 10.1007/s10067-013-2227-1 23483294

[6] Lerkvaleekul B, Vilaiyuk S. Macrophage activation syndrome: early diagnosis is key. Open Access Rheumatol. 2018;10:117. 10.2147/OARRR.S151013 PMC6124446 30214327

[7] Crayne CB, Albeituni S, Nichols KE, Cron RQ. The immunology of macrophage activation syndrome. Front Immunol. 2019;10:119. 10.3389/fimmu.2019.00119 PMC6367262 30774631

[8] Granata G, Didona D, Stifano G, Feola A, Granata M. Macrophage activation syndrome as onset of systemic lupus erythematosus: a case report and a review of the literature. Case Rep Med. 2015. 10.1155/2015/294041. PMC4439481 26064125

[9] Naveen R, Jain A, Muhammed H, Gupta L, Misra DP, Lawrence A, et al. Macrophage activation syndrome in systemic lupus erythematosus and systemic-onset juvenile idiopathic arthritis: a retrospective study of similarities and dissimilarities. Rheumatol Int. 2021;41(3):625–31. 10.1007/s00296-020-04763-6 33388903

[10] Hochberg MC. Updating the American College of Rheumatology revised criteria for the classification of systemic lupus erythematosus. Arthritis Rheum. 1997;40(9):1725. 10.1002/art.1780400928 9324032

[11] Miyakis S, Lockshin M, Atsumi T, Branch D, Brey R, Cervera R, et al. International consensus statement on an update of the classification criteria for definite antiphospholipid syndrome (APS). J Thromb Haemost. 2006;4(2):295–306. 10.1111/j.1538-7836.2006.01753.x 16420554

[12] Janka GE. Familial and acquired hemophagocytic lymphohistiocytosis. Eur J Pediatr. 2007;166(2):95–109. 10.1007/s00431-006-0258-1 17151879

[13] Hadchouel M, Prieur A-M, Griscelli C. Acute hemorrhagic, hepatic, and neurologic manifestations in juvenile rheumatoid arthritis: possible relationship to drugs or infection. J Pediatr. 1985;106(4):561–6. 10.1016/s0022-3476(85)80072-x 3981309

[14] Egües Dubuc CA, Uriarte Ecenarro M, Meneses Villalba C, Aldasoro Cáceres V, Hernando Rubio I, Belzunegui OJ. Hemophagocytic syndrome as the initial manifestation of systemic lupus erythematosus. Reumatol Clin. 2014;10(5):321–4. 10.1016/j.reuma.2013.09.004 24316336

[15] Henter JI, Horne A, Aricó M, et al. HLH-2004: diagnostic and therapeutic guidelines for hemophagocytic lymphohistiocytosis. Pediatr Blood Cancer. 2007;48(2):124–31. 10.1002/pbc.21039 16937360

[16] Fardet L, Galicier L, Lambotte O, Marzac C, Aumont C, Chahwan D, et al. Development and validation of the HScore, a score for the diagnosis of reactive hemophagocytic syndrome. Arthritis Rheumatol. 2014;66(9):2613–20. 10.1002/art.38690 24782338

[17] Deane S, Selmi C, Teuber SS, Gershwin ME. Macrophage activation syndrome in autoimmune disease. Int Arch Allergy Immunol. 2010;153(2):109–20. 10.1159/000312628 20407267

[18] Pak CC, Fidler IJ. Liposomal delivery of biological response modifiers to macrophages. Biotherapy. 1991;3(1):55–64. 10.1007/BF02175099 2009214

[19] Mizushima Y, Hamano T, Yokoyama K. Tissue distribution and anti-inflammatory activity of corticosteroids incorporated in lipid emulsion. Ann Rheum Dis. 1982;41(3):263–7. 10.1136/ard.41.3.263 PMC1000924 6896429

[20] Sen ES, Clarke SL, Ramanan AV. Macrophage activation syndrome. Indian J Pediatr. 2016;83(3):248–53. 10.1007/s12098-015-1877-1 26400031

[21] Nakagishi Y, Shimizu M, Kasai K, Miyoshi M, Yachie A. Successful therapy of macrophage activation syndrome with dexamethasone palmitate. Mod Rheumatol. 2016;26(4):617–20. 10.3109/14397595.2014.906053 24754272

[22] Pilania RK, Jindal AK, Johnson N, et al. Macrophage activation syndrome in children with Kawasaki disease: an experience from a tertiary care hospital in northwest India. Rheumatology (Oxford). 2021;60(7):3413–9. 10.1093/rheumatology/keaa715. 33221920

